# Using a novel medical arts program to integrate the art and science of medicine

**DOI:** 10.5116/ijme.523d.a0cf

**Published:** 2013-10-06

**Authors:** Joel D. Howell, Elissa Gaies, Sanjay Saint

**Affiliations:** 1 Department of Internal Medicine, University of Michigan Medical School, USA; 2 VA Ann Arbor Healthcare System, USA

Medicine is not fundamentally based on molecules, neither are patients nor their physicians merely collections of organs. To truly care for their patients, physicians need to engage with the complex lived experiences of both themselves and their patients. While medical educators are charged with guiding learners to become proficient with awe-inspiring new technologies and therapeutics for ever-more-sophisticated diagnosis and treatment, they must simultaneously ensure that their students embrace the essential, humanistic nature of healthcare. This is no easy task.Recognizing the necessary skills is only the beginning. How do we teach them? Although medical educators may do well at imparting the scientific knowledge necessary to deliver technically competent care, far too many physicians do not do a particularly good job at delivering humanistic patient care. While 78% of physicians think they provide compassionate care, only 50% of patients think physicians are comfortable discussing sensitive, emotional, or psychological needs, 51% think they consider the effect of the illness on them and their family, and 49% think they strive to understand their emotional needs.^1^ Our challenge as educators is to teach students how to provide patient care that moves beyond a century-long focus on the notion that natural science provides a generalizable model for diverse human beings to a new focus for care that incorporates the lived experiences of individual human beings.Perhaps the arts can help. Artists have long addressed issues central to human existence, such as joy and suffering, pestilence and prosperity, devotion and despair, and impending death. Artists have also grappled with the sorts of profound questions that can be central to the experience of illness, such as the nature of human relationships and life’s purpose. The arts can offer insight into the nexus between society and medicine with an intensity and fidelity that may be unavailable through other means.Some medical schools have used the arts to enhance students’ skills in observation and critical thinking^2^^-^^4^ and for confronting medical contradictions and complexities.^5^ Others have focused on re-evaluating concepts of moral absolutes,^6^ while still others have used the arts to better understand suffering and death.^6^^,^^7^ At the University of Michigan Medical School, we have created a new program that focuses neither on one specific skill nor on a single type of art, but seeks instead to use a wide range of arts as a way to enhance medical students’ and house officers’ ability to provide high-quality, humanistic clinical care. While in our program we often attend to artistic works that focus explicitly on health and illness, we also believe that the essential humanity we seek to foster is not so limited and thus include art that has no explicit connection to health care. We include a diverse group of artistic media, including visual arts, music, theater, dance, and literature. The Medical Arts Program collaborates with several local artistic organizations, including a performing arts organization, the University Musical Society, as well as the University of Michigan Museum of Art, the University of Michigan School of Music, Theater and Dance, and the University of Michigan Master of Fine Arts (MFA) Program.Integral to the Medical Arts Program is the opportunity for learners to analyze intensively each event with content experts and visiting artists. Whenever possible, artists and performers spend time observing the process of care in clinical settings before meeting with learners. This experience enables them more effectively to relate their insights from the arts to learners and to clinical practice.The Program offers about eight events annually. Attendance is about 30-40 learners per event. Since its inception in 2009, over 250 unique learners have participated in 24 events. Recent events include a performance of the award-winning, innovative opera Einstein on the Beach followed by a discussion with one of the artists; a performance by the world-renowned chamber music ensemble The Takács Quartet linked with a conversation with the members of the quartet; a reading and conversation with the Pulitzer-prize winning poet Rae Armantrout, together with University of Michigan MFA students; and a tour of the internationally renowned Ghanaian-born El Anatsui’s recent retrospective at the University of Michigan Museum of Art led by an expert in West African art. Our website contains a full list of past events as well as other program information such as event evaluations, funding, and leadership.

The University of Michigan Health System Institutional Review Board granted this study “exempt” status because the evaluation of the Medical Arts Program is being conducted in “an established, commonly accepted educational setting that involves normal educational practices and the research is on the effectiveness of instructional techniques.” We are using a mixed-methods approach to formally evaluate the impact of the Program. This includes parallel evaluations of learners who are attending events and a comparison group of learners who are not participating in the Medical Arts Program. We also asked learners to self-evaluate each session. While the formal, comparative analysis is still incomplete, when asked if they would like to attend another Medical Arts event, 96% of learners strongly agreed. A selection of learners’ reflections is shown in Table 1.

**Table 1:** Learners’ reflections on medical arts events

Medical arts event	Learners’ reflections
Learners felt that a visit to the University of Michigan Museum of Art was useful in helping them deal with the ambiguity present in medical care.	“As a resident, I frequently feel one-dimensional. The students' discussion of the works of art was very subjective. In the hospital and clinic, we largely deal in objective matters. It's nice to be reminded that reasonable people can have disparate qualitative views.”
During a painting workshop learners had the opportunity to create their own art.	“Creating art is about telling a story - and taking care of patients is about listening to their subjective experiences and trying to put together our own ‘story’ to explain their signs/symptoms.”
Learners attended a concert followed by a discussion with the innovative musicians Gabriel Kahane and yMusic.	“Very valuable to see the combination of modern classical music and rock music - how one genre can inform and add to the other. A reminder of the importance of interdisciplinary work, which is especially important to us in carrying out effective patient care.”
Learners watched Einstein on the Beach, a 4.5 hour opera (without intermission).	“A lot of what we do is very repetitive, ex. ordering labs, checking vital signs, writing progress notes, and it is very easy to lose focus and attention…When we take time and energy to be aware of what we are doing, medical practice becomes more refined, cost effective, safe, and patient-centered.”
Learners were asked to select a work in the contemporary art gallery that they either didn’t understand or didn’t like – just as physicians provide care to some patients they don’t understand, or they don’t like. An especially engaging piece was a work by the artist Gonzalez-Torres. His piece consisted of only two extension cords draped over a nail with two lit light bulbs. While the initial reaction to this work of art was incomprehension, after the curator-led discussion learners grasped that the piece symbolized the life and love that the artist shared with his partner who had AIDS (the intertwined cords, and the bulbs resting on each other) as well as the finitude of life (one light will burn out before the other).	“[This instillation]...revealed very raw human emotion, a type of emotion not often expressed to outsiders. As physicians, we are allowed deep into the human condition of near strangers on a daily basis.”

Not just exploring the lived experiences of others, the Medical Arts Program enables students to examine their own lived experiences, using the arts to understand their own vulnerability and values as well as to be more open and comfortable discussing this with others. In addition, these events have served as a catalyst for innovative cross-campus interactions and have helped to break down the artificial barriers that all too often separate the world of healthcare from the rest of the university, especially the world of the arts. In addition they have raised awareness of the importance of the arts for healthcare, and of the importance of healthcare for scholarship throughout the university.In 1927 Frances W. Peabody noted that “the treatment of a disease may be entirely impersonal; the care of a patient must be completely personal.”^8^ Through this exposure to the diversity of artistic media and analyses with content experts our learners are exploring new ways to incorporate Peabody’s wisdom into their practice - to care for their patients rather than merely to treat their diseases. Over the last century we have made great strides in incorporating lessons from science into medical education. Perhaps there is also value to be gained from the arts.

## Conflict of Interest

The authors declare that they have no conflict of interest.
